# Identification of FABP7 as a Potential Biomarker for Predicting Prognosis and Antiangiogenic Drug Efficacy of Glioma

**DOI:** 10.1155/2022/2091791

**Published:** 2022-06-24

**Authors:** Liubing Hou, Huandi Zhou, Yanqiang Wang, Junling Liu, Dongdong Zhang, Yuehong Li, Xiaoying Xue

**Affiliations:** ^1^Department of Radiotherapy, The Second Hospital of Hebei Medical University, Shijiazhuang, 050000 Hebei Province, China; ^2^Department of Central Laboratory, The Second Hospital of Hebei Medical University, Shijiazhuang, 050000 Hebei Province, China; ^3^Department of Pathology, The Second Hospital of Hebei Medical University, Shijiazhuang, 050000 Hebei Province, China

## Abstract

**Objective:**

Glioma is a common malignant tumor of the central nervous system with extremely poor prognosis. An efficient molecular marker for diagnosis and treatment is urgently needed. Fatty acid binding protein 7(FABP7), which regulates intracellular lipid metabolism, is highly expressed in nervous system tumors, but its prognostic value remains undetermined. The present study investigated the relationship between FABP7 expression and prognosis in glioma patients by bioinformatics analysis, as well as immunohistochemically evaluating the effect of FABP7 expression on the efficacy of antiangiogenic drugs.

**Methods:**

Gene expression and clinical data on patients with glioma were collected from the China Glioma Genome Atlas (CGGA) database, The Cancer Genome Atlas (TCGA), and the Gene Expression Omnibus (GEO) databases. Levels of FABP7 expression and their association with the clinicopathologic characteristics of glioma patients were analyzed in the CGGA database. The relationships between FABP7 expression and clinical findings, such as survival and prognostic, were determined and used for nomogram construction. Mechanisms of action of FABP7 were assessed using GSEA software. FABP7 expression in the tissues of glioma patients treated with apatinib was evaluated immunohistochemically.

**Results:**

FABP7 was highly expressed in glioma samples, with higher FABP7 expression associated with poorer patient prognosis and more advanced clinicopathological features. Bioinformatics analysis, including survival, receiver operating characteristic curve, and univariate and multivariate Cox analyses, showed that FABP7 was independently prognostic of outcomes in glioma patients. GSEA analysis showed that FABP7 was associated with angiogenesis, with FABP7 having correlation coefficients > 0.4 with seven factors in the angiogenic pathway, POSTN, TIMP1, PDGFA, FGFR1, S100A4, COL5A2, and STC1, and the expression of these factors related to patient prognosis. Immunohistochemistry showed that FABP7 expression was higher in glioma patients with poor survival after apatinib treatment.

**Conclusions:**

High FABP7 expression is associated with poor prognosis in glioma patients. FABP7, which is important for glioma angiogenesis, may serve as an independent prognostic predictor in glioma patients.

## 1. Introduction

Glioma is the most common primary malignant tumor of the central nervous system, accounting for about 70% of all primary malignant brain tumors [1]. The highly invasive and aggressive nature of these tumors makes them difficult to completely eliminate by current treatment strategies, such as surgery, radiotherapy, and chemotherapy, leading to poor patient prognosis [2]. Biomarkers are needed to accurately predict patient and responses to individualized treatment modalities. Bioinformatics analysis has been used to identify oncogenes with various biomarkers for cancer diagnosis and prognosis identified including in patients with glioma. Although several molecular markers were shown to be prognostic, none of the biomarkers identified to date have become targets for therapy [3–5]. Identification of new target biomarkers may improve the ability to treat glioma.

During carcinogenesis, cells acquire the ability to proliferate uncontrollably, requiring reprogramming of cancer cell metabolism. This reprogramming provides cells sufficient metabolic plasticity to meet high energy requirements. Lipid metabolism disorders have been reported to affect the survival of cancer cells. Fatty acid-binding proteins (FABPs) are a multigene family of low molecular weight (14–15 kDa) proteins that regulate fatty acid uptake and the intracellular formation of lipid droplets (LDs), suggesting that FABPs are central regulators of lipid metabolism and energy homeostasis [6]. In cancer, LDs protect malignant cells from reactive oxygen species (ROS) and support their survival during reoxygenation after hypoxia [7], suggesting that methods of regulation of fatty acid transport and storage may enhance the survival of cancer cells [8, 9]. Adipose tissue and free fatty acids play important roles in the survival, proliferation, and migration of cancer cells [10–12].

FABPs can act as prognostic markers for survival in patients with various types of tumors. For example, FABP2 is highly expressed in gastric cancer [13], whereas high FABP3 expression has been associated with poor prognosis in patients with hepatocellular carcinoma (HCC) [14, 15] and esophageal cancer [16, 17]. FABP4 is an important predictor of poor prognosis in patients with stomach adenocarcinoma [18], colorectal cancer [19], and cervical cancer [20], whereas FABP5 is an independent prognostic factor in patients with uveal melanoma [21]. In addition, the levels of FABP6 mRNA were found to be higher in HCCs than in adjacent normal liver tissues [22]. FABP1 and FABP6 are strongly expressed in colorectal cancer tissues, with overexpression of FABP6 protein was found to be associated with poorer overall survival (OS) in colorectal cancer patients [23]. The levels of FABP9 was found to be higher in breast cancer samples than in adjacent normal breast tissues [24].

FABP7, also called brain-type FABP (B-FABP) or brain lipid-binding protein (BLBP), has been found to bind long-chain fatty acids and dissolve long-chain unsaturated fats [6]. In addition, FABP7 was shown to absorb fatty acids and transport them into cells, as well as to regulate the balance of lipid metabolism in cells [6]. FABP7 is highly expressed in many types of tumors, especially in those of the nervous system, as FABP7 has been shown essential for the establishment of a radial glial fiber system [11, 12]. To date, however, the role of FABP7 in the prognosis of glioma has not been clearly demonstrated.

Although tumor growth depends on angiogenesis [25], drugs targeting blood vessels in tumors generally usually have limited effect on patient survival improvement [26]. The low efficacy of those agents may be due to the development of tumor resistance to antiangiogenic drugs [27–29]. Antiangiogenic drugs can induce hypoxia and lipolysis, limit glucose supply, and trigger lipid-dependent metabolic reprogramming, resulting in increased free fatty acids and cancer cell proliferation and switching the glucose-dependent metabolism of cells to lipid-dependent metabolism. Inhibition of fatty acid oxidation may enhance the therapeutic effect of antiangiogenic drugs [30]. FABP7 has been shown to be involved in lipid metabolism in various tumors, particularly gliomas [7, 31], suggesting that the abnormal expression of FABP7 may influence the prognosis of patients with glioma and their response to treatment with the antiangiogenic agent apatinib. Therefore, the present study analyzed the levels of expression of FABP7 in glioma and the relationship between FABP7 expression and clinicopathological features of glioma patients in the CGGA and TCGA databases. The effect of FABP7 on treatment with antiangiogenic drugs was assessed by immunohistochemistry. The results of this study showed that FABP7 was overexpressed in glioma and that higher FABP7 expression was associated with poor patient prognosis and poor response to apatinib. These results suggested that FABP7 may act as a potential prognostic biomarker and may be a promising target in glioma patients being treated with apatinib.

## 2. Materials and Methods

### 2.1. Data Collection

Gene expression data and corresponding clinical data were obtained from the Chinese Glioma Genome Atlas (CGGA) database (http://www.cgga.org.cn/). A total of 1,018 patients in two datasets DataSet ID: mRNAseq_325 and mRNAseq_693, consisting of 325 and 693 samples, respectively, and 20 nonglioma brain tissues were downloaded. For validation, 592 glioma samples were collected from The Cancer Genome Atlas (TCGA) database (https://portal.gdc.cancer.gov/). The GSE4290 dataset, containing 153 glioma samples and 23 normal samples, and the GSE7696 dataset containing 80 glioma samples and 4 normal samples, were obtained from the Gene Expression Ominibus (GEO) database (https://www.ncbi.nlm. http://nih.gov/geo/).

### 2.2. Expression Analysis

FABP7 mRNA expression in glioma and normal brain tissues were downloaded from the CGGA and TCGA databases, and GSE4290 and GSE7696 datasets were analyzed using GraphPad Prism 8.0 statistical software. FABP7 protein levels in normal and glioma tissues downloaded from the Human Protein Atlas (HPA) (https://www.proteinatlas.org/) and in various cancers downloaded from the TIMER website (https://cistrome shinyapps.io/timer/) were analyzed by immunohistochemistry.

### 2.3. Correlations between FABP7 Expression and Clinicopathologic Characteristics

The median levels of expression of FABP7 in glioma tissues from the CGGA dataset were determined, and the patients were divided into those with high and low FABP7 expression based on the medium. The survival curves of patients with different FABP7 expression levels were drawn using the “survival” and “survminer” packages in R software. Receiver operating characteristic (ROC) curves of the relationship between FABP7 expression and 1-, 3-, and 5-year survival were determined by the Kaplan–Meier method using the “survival ROC” software package. The predictive value of FABP7 expression at a significance level of *p* < 0.001 was assessed by univariate and multivariate Cox analyses. Survival and ROC curves were also assessed in the TCGA and CGGA mRNAseq_301 datasets. Individual prognostic signatures for 1-, 2-, and 3-year survival were utilized to construct a nomogram in CGGA using the “survival” and “rms” packages of R software. Calibration curves were plotted to evaluate the concordance between actual and predicted survival. The relationships between FABP7 expression and clinicopathologic characteristics were analyzed using the “beeswarm” package in R software.

### 2.4. Analysis of Differentially Expressed Genes (DEGs) and Enrichment of Significant Pathways and Gene Set Enrichment Analysis (GSEA)

DEGs in samples from the CGGA dataset with high and low expression of FABP7 including significantly upregulated and downregulated genes, defined as those with an adjusted *p* value < 0.05 and absolute log2 fold change (FC) > 1, were screened using the limma R package. To further explore the function and mechanism of action of FABP7, GO enrichment analysis was performed using Metascape (http://metascape.org/), following by gene set enrichment analysis (GSEA). A normalized enrichment score (NES) > 1 and a false discovery rate < 0.05 were considered meaningful.

### 2.5. Analysis of Correlation Coefficients between Angiogenic Pathway Factors and FABP7

The correlations between the core genes of the angiogenic pathway obtained by GSEA and FABP7 were analyzed using the Sangerbox online tool (http://sangerbox.com/), with genes having a correlation coefficient > 0.4 with FABP7 identified. The association between these factors and was determined by Gene Expression Profiling Interactive Analysis (GEPIA, http://gepia.cancer-pku.cn/), with high expression associated with poor prognosis.

### 2.6. Specimen Preparation

Glioma specimens from six patients who were treated with were collected from Radiotherapy Department, and specimens from 10 patient tissues who underwent neurosurgery were collected from the Neurosurgery Department of the Second Hospital of Hebei Medical University. This research was approved by the Ethics Committee of the Second Hospital of Hebei Medical University. All patients provided written informed consent.

### 2.7. Immunohistochemical Analysis

Two pathologists evaluated three representative visual fields in each tumor specimen at magnifications of 10∗20 times. The percentage of positive cancer cells in each tumor specimen and their staining intensity were evaluated. The percentage of positive cancer cells was scored as 0 (≤5%), 1 (6–25%), 2 (26–50%), 3 (51–75%), or 4 (76–100%); and staining intensity was scored as 0 (no staining), 1 (light staining), 2 (moderate staining), or 3 (deep staining). The final score of each specimen was calculated by multiplying the staining degree score and the staining cell percentage. Discrepancies in scores were resolved by the consensus of the two pathologists.

### 2.8. Western Blot

Proteins were extracted from glioma tissues using RIPA buffer (Beyotime, Shanghai, China) with protease inhibitor (Solarbio, China) and their concentrations measured using BCA kit (Solarbio). Aliquots containin 20 *μ*g protein were separated on 10% SDS-PAGE gels and transferred to PVDF membranes (Millipore, MA, USA). The membranes were incubated with 5% skimmed milk for 1 hour and incubated overnight at 4°C with primary antibodies against FABP7 (1 : 1000, 51010-1-AP, Proteintech, China) and GAPDH (1 : 10000, 10494-1-AP, Proteintech) The membranes were washed three times for 10 minutes each time with TBST, incubated with secondary antibody at room temperature for 1 hour, and again washed three times with TBST. Images were developed using an ECL western blot kit (biosharp, China), and the density of each band was measured using Image lab software (Bio-Rad, USA).

## 3. Results

### 3.1. Sample Characteristics

**Figure 1:**
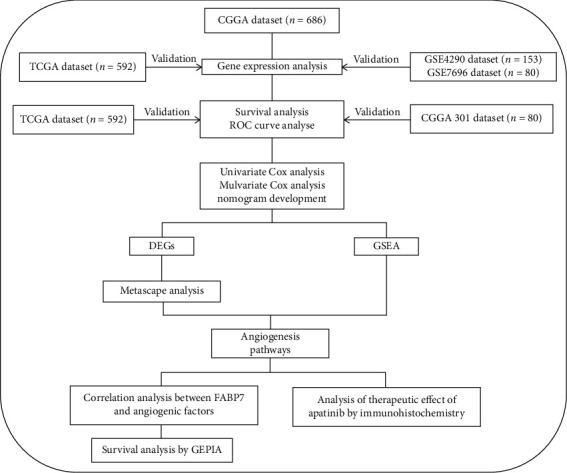
Workflow. Abbreviations: CGGA: Chinese Glioma Genome Atlas; TCGA: The Cancer Genome Atlas; GEO: Gene Expression Omnibus; ROC: receiver operator characteristic; DEGs: differentially expressed genes; GSEA: gene set enrichment analysis; GEPIA: Gene Expression Profiling Interactive Analysis.

The workflow of this study is shown in Figure 1. After screening out the invalid information, clinical and mRNA expression data were obtained from 686, 592, 153, and 80 samples of patients with glioma in the CGGA, TCGA, GSE4290, and GSE7696 datasets, respectively. The clinical data of in the CGGA dataset included PRS type, tumor grade, age, gender, OS, radiotherapy status, chemotherapy status, IDH mutation status, MGMT methylation status, and 1p19q codeletion status. Data in the TCGA dataset included only tumor grade, age, gender, OS, IDH mutation status, and 1p19q codeletion status. Detailed clinical information on these glioma patients are shown in Table 1.

### 3.2. FABP7 mRNA and Protein Expression in Glioma

**Figure 2:**
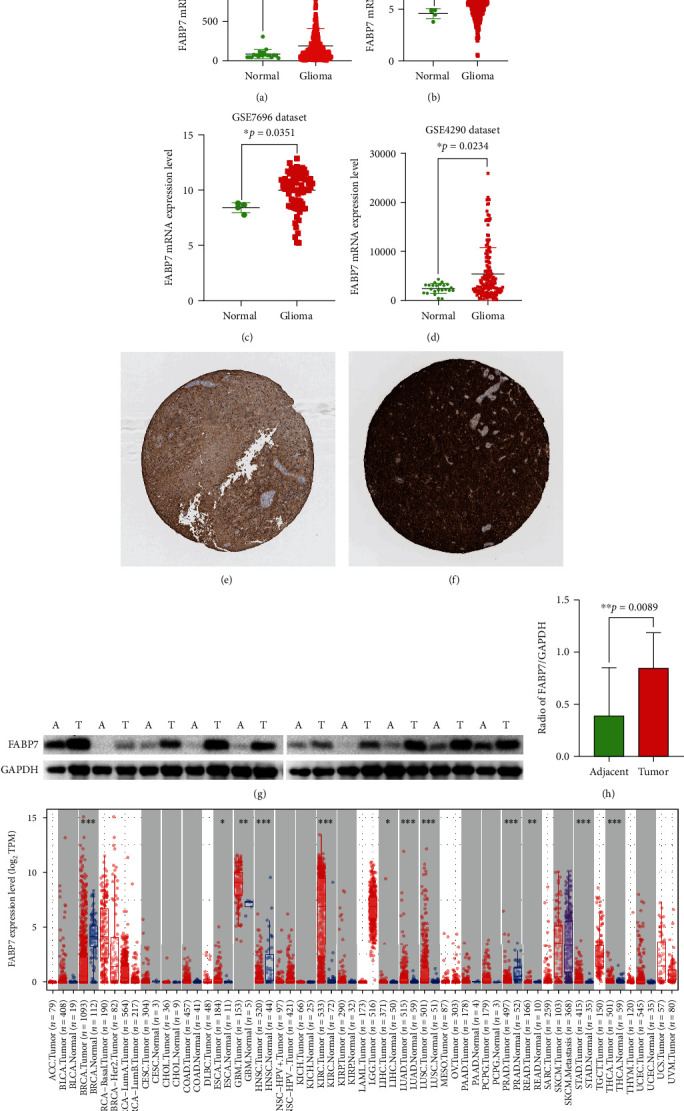
Gene expression of FABP7 in glioma and normal tissues. Comparison of FABP7 mRNA expression levels between glioma and normal tissues. (a) CGGA dataset. (b) TCGA dataset. (c) GSE7696 dataset. (d)GSE4290 dataset. (e) Protein levels of FABP7 in normal tissue by immunohistochemistry based on the HPA website (staining: medium; intensity: moderate; quantity: >75%, patient id 1537). (f) Protein levels of FABP7 in glioma tissue by immunohistochemistry based on HPA website (staining: high; intensity: strong; quantity: >75%, patient id 2790). (g, h) Expression of FABP7 in 10 pairs of glioma and adjacent tissues tested by western blot. (i) Expression of FABP7 in various cancers based on TIMER2.0. T: tumor tissue; A: adjacent tissue.

Analysis of FABP7 mRNA expression in the CGGA, TCGA, GSE4290, and GSE7696 datasets showed that the expression of FABP7 was significantly higher in glioma samples than in normal tissues (Figures 2(a)–2(d)). In addition, evaluation of data from the HPA website showed that FABP7 protein levels were markly higher in glioma (Figure 2(f)) than in normal (Figure 2(e)) tissues. Similar results were observed following western blot analysis of 10 pairs of glioma and adjacent normal tissues (Figures 2(g) and 2(h), *p* = 0.0089). Moreover, evaluation of the TIMER website showed that FABP7 was highly expressed in many tumors, especially in glioma (Figure 2(i)).

### 3.3. Analysis of FABP7 Expression as Prognosis of Survival in Glioma Patients

**Figure 3:**
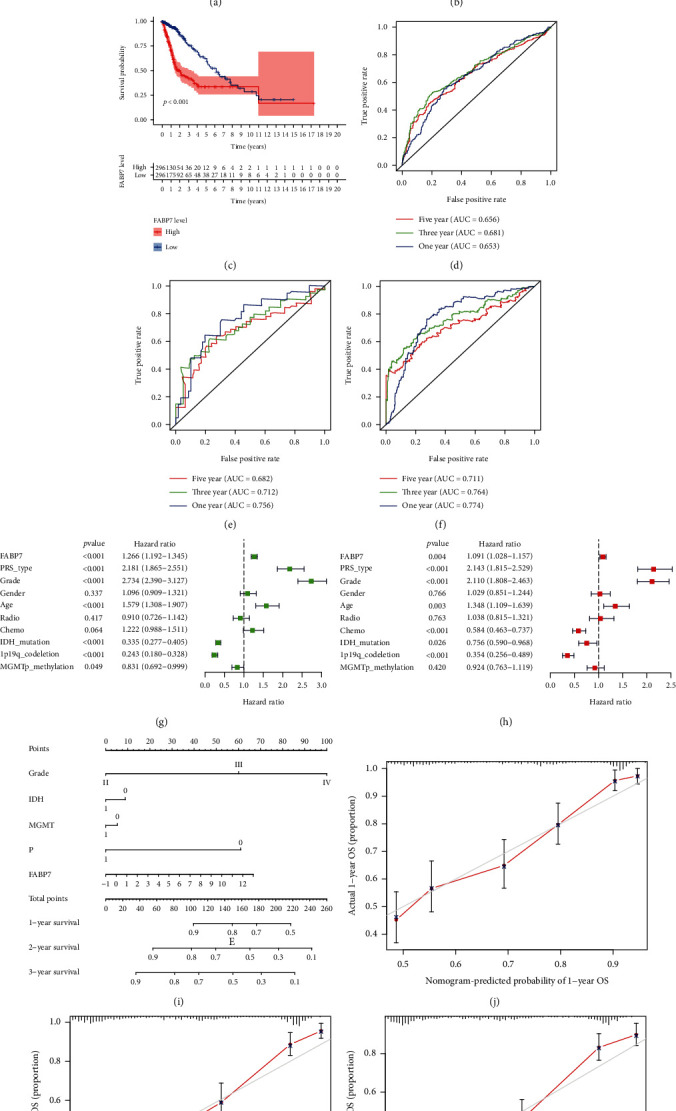
The relationship between FABP7 and prognosis of glioma patients by bioinformatics analysis based on the CGGA dataset and TCGA dataset and independent prognostic analysis and development of nomogram based on the CGGA dataset. Survival analysis in the high FABP7 and low FABP7 groups in glioma patients. (a) Based on the CGGA dataset (*n* = 686). (b) Based on the CGGA mRNAseq_301 dataset (*n* = 80). (c) Based on the TCGA dataset (*n* = 592). The ROC curve of FABP7. (d) Based on the CGGA dataset (*n* = 686). (e) Based on CGGA mRNAseq_301 dataset (*n* = 80). (f) Based on the TCGA dataset. (*n* = 592).  Figure 4 (*n* = 686). (g) Univariate analysis of FABP7. (h) Multivariate analysis of FABP7. (i) Prognostic nomogram to predict the survival of glioma patients. (j–l) Nomogram calibration curves for predicting survival at 1, 2, and 3 years in the CGGA training cohort.

Kaplan–Meier survival analysis of clinical data from the CGGA dataset showed that high FABP7 expression was significantly associated with reduced survival in glioma patients (*p* < 0.001, Figure 3(a)). These results were validated by analyses of the TCGA and CGGA mRNAseq_301 datasets (Figures 3(b) and 3(c)). ROC curve analysis of the CGGA dataset showed that the areas under the ROC curves (AUCs) for 1-, 3-, and 5-year survival were 0.653, 0.681, and 0.656, respectively (Figure 3(d)). Similarly, the AUCs for 1-, 3-, and 5-year survival were 0.756, 0.712, and 0.682, respectively, in the CGGA mRNAseq_301 dataset and 0.774, 0.764, and 0.711, respectively, in the TCGA dataset (Figures 3(e) and 3(f)). These results indicated that the level of FABP7 expression could be used as a predictive marker for 1-, 3-, and 5-year survival in patients with glioma.

Univariate and multivariate Cox regression analyses of the CGGA dataset were performed to determine whether FABP7 is an independent prognostic indicator of survival in glioma patients. Univariate analysis indicated that high FABP7 expression, PRS type, tumor grade, age, chemotherapy status, IDH mutation status, and 1p19q codeletion status were associated with reduced OS in patients with glioma (Figure 3(g)). Multivariate analysis showed that the high FABP7 expression, PRS type, tumor grade, age, chemotherapy status, and MGMT methylation status were independently predictive of OS in these patients (Figure 3(h)). Thus, these results indicated that FABP7 is independently prognostic of survival in patients with in glioma.

**Figure 4:**
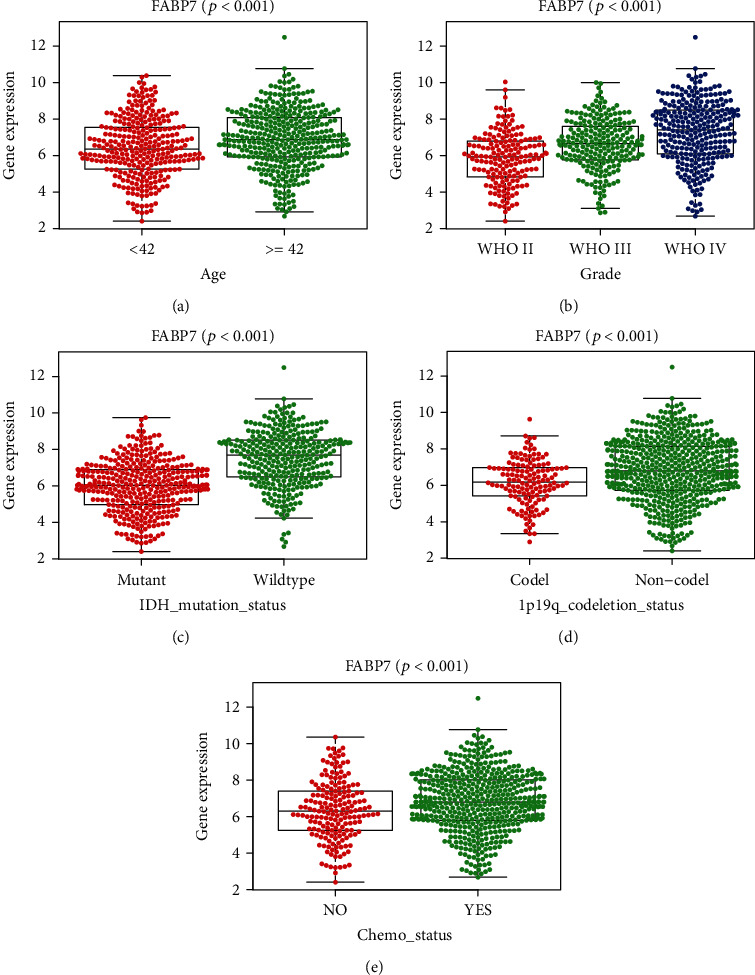
Correlation analysis between FABP7 expression and clinical characteristics using the CGGA dataset in glioma patients. Differential expression of FABP7 was significantly related to (a) age (<42, *n* = 307; and ≥42, *n* = 379), (b) grade (WHO II, *n* = 177; WHO III, *n* = 226; and WHO IV, *n* = 283), (c) IDH mutation status (mutant, *n* = 371; wildtype, *n* = 315), (d) 1p19q codeletion status (codel, *n* = 141; noncodel, *n* = 545), and (e) chemo status (yes, *n* = 501; no, *n* = 105).

To better predict the prognosis of patients with glioma, a nomogram was constructed using FABP7 expression level, tumor grade, IDH mutation status, MGMT methylation status, and 1p19q codeletion status. The level of FABP7 expression was found to be the dominant factor in the predictive ability of the nomogram (Figure 3(i)). Analysis of correction curves showed that the actual and predicted survival matched well at 1, 2, and 3 years (Figures 4(j)–4(l)). These findings suggest that FABP7 may be a biomarker for predicting the survival of glioma patients and that increased FABP7 expression was associated with poor prognosis.

### 3.4. Relationship between FABP7 Expression and Clinical Characteristics in Patients with Glioma

Further analysis of the association of FABP7 expression with clinicopathologic characteristics of patients in the CCGA dataset showed that the level of FABP7 expression in patients with glioma correlated significantly patient age and tumor grade (*p* < 0.001, Figures 4(a) and 4(b)). The expression of FABP7 was lower in patients with 1p19q codeletion and in those IDH1 mutation (*p* < 0.001, Figures 4(c) and 4(d)) but was higher in patients who received chemotherapy (*p* < 0.001, Figure 4(e)).

### 3.5. DEGs and GSEA of FABP7

**Figure 5:**
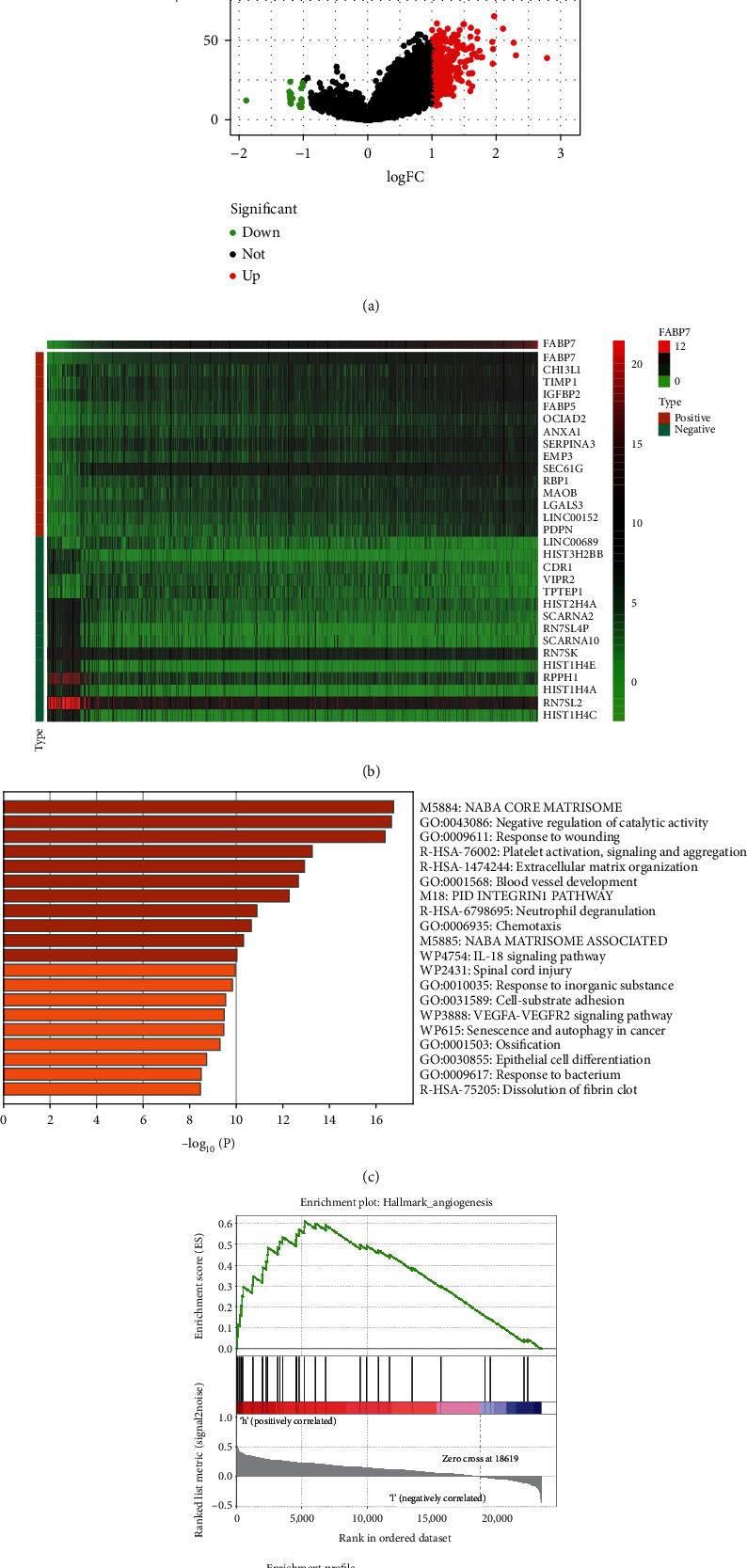
DEGs and GSEA enrichment analysis of FABP7. (a) Volcano plot of DEGs with log2 − fold change (log2FC) > 1 were shown in red; DEGs with (log2FC) < −1 were shown in green (*p* < 0.05). (b) Heatmap of the top 15 genes positively and negatively associated with FABP7. (c) Heatmap of enriched terms across DEGs in Metascape analysis. (d) Enrichment plot of the angiogenesis pathway from GSEA.

To further explore the function of FABP7, DEGs were screened using Sangerbox software under the condition of |log2FC < 1|. The 331 DEGs screened included 312 upregulated and 19 downregulated genes (Figure 5(a)). A heatmap of the top 15 up- and downregulated genes is showed in Figure 5(b). Metascape analysis of these DEGs showed that one of the biological processes significantly enriched in glioma was blood vessel development (Figure 5(c)). In addition, GSEA software identified pathways involved in glioma, with the angiogenesis pathway being significantly enriched (NES = 1.66, *P* = 0.039, FDR = 0.092; Figure 5(d)). These results suggest that FABP7 may influence the survival of glioma patients through angiogenesis.

### 3.6. Correlation between FABP7 and Angiogenic Factors

**Figure 6:**
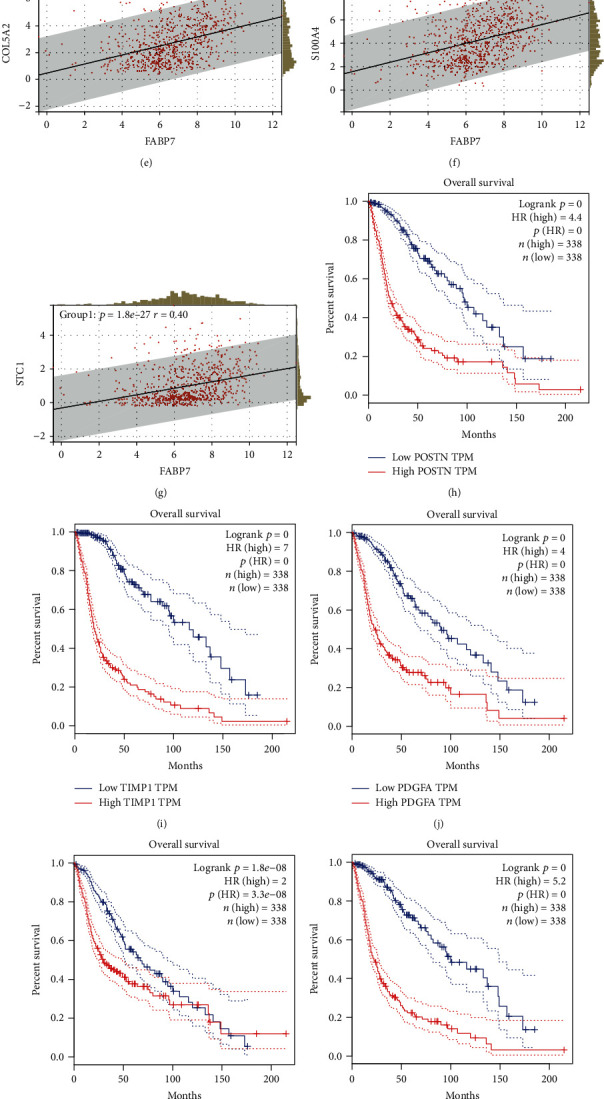
Correlation between FABP7 and angiogenic factors including POSTN, TIMP1, PDGFA, FGFR1, S100A4, COL5A2, and STC1 (a–g). Prognostic analysis of those factors (h–n).

Gene correlation analysis showed that FABP7 expression correlated significantly with the levels of expression of the angiogenic factors POSTN, TIMP1, PDGFA, FGFR1, S100A4, COL5A2, and STC1 (Figures 6(a)–6(g)), indicating that FABP7 is related to the angiogenesis pathway. Survival analysis showed that higher expression of each of the seven factors was accompanied by poorer survival outcomes in patients with glioma (Figures 6(h)–6(n)).

### 3.7. Association of FABP7 Expression with Apatinib Treatment of Glioma Patients

**Figure 7:**
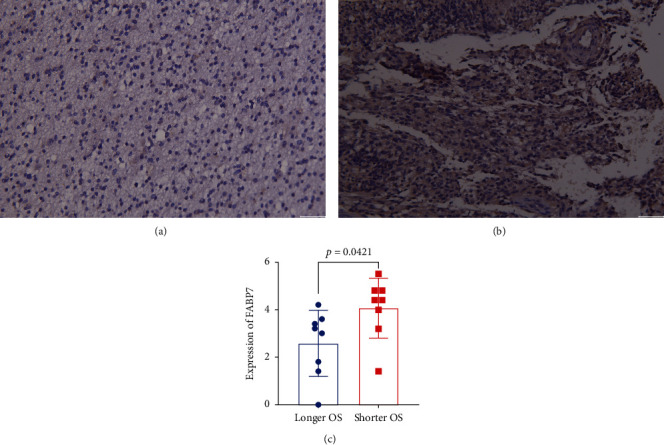
Immunohistochemical of glioma patients treated with apatinib. (a) Low expression of FABP7. (b) High expression of FABP7. (c) Numerical value of FABP7 expression.

Glioma and accompanying normal tissue samples selected from sixteen glioma patients who were treated with apatinib were assessed immunohistochemically using antibodies to FABP7. The patients were divided into two groups according to the median OS. The results indicated that FABP7 was more highly expressed in patients with shorter than longer OS (Figures 7(a)–7(c)), suggesting that FABP7 might overcome effects of antiangiogenic drugs by promoting angiogenesis.

## 4. Discussion

Cancer cells can grow in challenging environments, including in hypoxic and nutrient-deficient tumor microenvironments. Adipocytes can be activated by cancer cells, inducing lipolysis and the secretion of fatty acids, including triglycerides. These secreted fatty acids can be transported to cancer cells by large number of fatty acid transporters. Cancer-associated fibroblast cells are also involved in lipid secretion, leading to cancer cell catabolism and lipid signal transduction. Abnormal lipid metabolism, such as increased fatty acid oxidation, can provide survival advantages for tumors, enhancing their resistance to chemotherapy and radiotherapy [30]. Therefore, the abnormal expression of proteins associated with lipid metabolism may play an important role in the occurrence and development of tumors.

FABP7, a member of the FABPs protein family, binds to long-chain fatty acids and dissolves long-chain unsaturated fats, which are absorbed and transported into cells to regulate lipid metabolism. FABP7 is expressed in many types of tumors, including brain, breast, colorectal, and prostate cancers, and is likely to play important roles in various cancers. FABP7 has been associated with response to chemotherapy and may be a potential biomarker predicting responses of breast cancer to neoadjuvant chemotherapy [32]. In addition, increased levels of FABP7 have been associated with reduced survival and a higher incidence of brain metastases in patients with HER2+ breast cancer [10]. FABP7 is overexpressed and promotes cell proliferation and survival in melanoma and colon cancer [33, 34]. Higher FABP7 expression in clear cell renal cell carcinoma was shown to significantly correlate with distant metastasis and poor prognosis, suggesting that FABP7 could be a potential tumor marker and therapeutic target in patients with these tumors [35, 36]. In addition, the expression of both Notch1 and FABP7 had important prognostic value in patients who underwent resection of tracheobronchial adenoid cystic carcinomas [37].

FABP7 expression is closely related to the occurrence and development of glioma. For example, high expression of FABP7 was found to enhance the proliferation and migration of glioblastoma (GBM) cells and to be associated with poor patient prognosis [12]. FABP7 was observed to regulate the metabolism of acetyl-CoA by interacting with ACLY in the nucleus [38] and to protect astrocytes from ROS toxicity through lipid droplet formation [7]. FABP3 and FABP7 were found to induce significant lipid droplet accumulation under hypoxic conditions, suggesting that these proteins play important roles in tumorigenesis in vivo [11].

Although the above studies indicate that FABP7 plays an important role in the development of glioma, few studies have evaluated the potential prognostic effect of FABP7 in glioma or its mechanism of action in these tumors. The present study evaluated the potential role of FABP7 in glioma by analyzing its expression in a large number of human gliomas. Analysis of the levels of FABP7 mRNA in the CGGA, TCGA, and GEO datasets showed that FABP7 mRNA was overexpressed in patients with glioma. In addition, HPA results and western blotting of paired tumor and normal tissue samples from 10 patients with glioma showed that the level of FABP7 protein was higher in glioma than in normal tissues. Kaplan–Meier survival analysis of patients in the CGGA (*n* = 686), CGGA mRNAseq_301 (*n* = 80), and TCGA (*n* = 592) datasets showed that high expression of FABP7 was associated with poor patient prognosis. The AUCs for 1-, 3-, and 5-year survival were 0.653, 0.681, and 0.656, respectively, for the CGGA dataset; 0.756, 0.712, and 0.682, respectively, for the CGGA mRNAseq_301 dataset; and 0.774, 0.764, and 0.711, respectively, for the TCGA dataset. EGFR activation may induce nuclear FABP7 expression, enhancing the migration of GBM cells, and an analysis of tissue samples from 123 patients with GBM showed that FABP7 overexpression correlated with shorter survival [39]. Moreover, overexpression of FABP7 promoted invasion and migration of GBM cell lines, with OS being longer in 17 GBM patients expressing lower levels of FABP7 than in 16 patients expressing higher levels of FABP7 [12]. Similarly, the present study showed that FABP7 was an independent prognostic marker for survival in patients with glioma. In addition, FABP7 expression correlated significantly with age, tumor grade, chemotherapy status, 1p19q codeletion, and IDH1 mutation status The potential mechanism of action of FABP7 and the relationship between FABP7 expression and glioma were further assessed by GSEA enrichment analysis which found that FABP7 was associated with angiogenesis. Immunohistochemical results showed that FABP7 expression was significantly higher in glioma patients with poor prognosis. High level of FABP7 may enhance tumor progression by inducing resistance to antiangiogenic drugs.

The present study has several limitations, including the small number of samples included for clinical validation. Moreover, additional studies are needed to determine the mechanism that regulated FABP7 expression.

## 5. Conclusions

The present study found that high expression of FABP7 correlated with poor prognosis in patients with glioma. FABP7 may be prognostic of survival outcomes in patients with glioma and may influence tumor progression by promoting tumor angiogenesis.

## Data Availability

The data sets generated during and/or analyzed during the current study are presented in the main file. Additional data are available from the corresponding author on reasonable request.
